# Towards multi-modal, multi-species brain atlases: part one

**DOI:** 10.1007/s00429-023-02656-5

**Published:** 2023-05-25

**Authors:** Rogier B. Mars, Nicola Palomero-Gallagher

**Affiliations:** 1 Wellcome Centre for Integrative Neuroimaging, Centre for fMRI of the Brain (FMRIB), Nuffield Department of Clinical Neurosciences, John Radcliffe Hospital, University of Oxford, Oxford, OX3 9DU UK; 2 Donders Institute for Brain, Cognition and Behaviour, Radboud University Nijmegen, AJ 6525 Nijmegen, The Netherlands; 3 Institute of Neuroscience and Medicine (INM-1), Research Centre Jülich, 52425 Jülich, Germany; 4 C. & O. Vogt Institute for Brain Research, Heinrich-Heine-University, 40225 Dusseldorf, Germany

Neuroanatomical knowledge is a fundamental component of neuroimaging analyses, since it enables researchers to interpret their findings in the context of the underlying cytoarchitectonic, molecular and connectional segregation of the brain. The digital era of neuroscience and current open science practices have resulted in the generation and availability of numerous datasets reflecting different aspects of the brain’s structural and functional segregation. This pertains not only the human brain, or that of non-human primates or rodents (Grandjean et al. 2023; Milham et al. 2018), the most commonly used animal models in neuroscience, but also the brains of other animal models, such as the mini pig (Bjarkam et al. 2017) and squirrel monkey (Orset et al. 2023), and even wider ranges of species across the mammalian class (Tendler et al. 2022; Suarez et al. 2022).

These developments, together with the increased awareness for the relevance of interpreting functional data in the framework of the brain’s structural segregation, have resulted in a plethora of new maps which have been acquired using very different methods, and which depict different levels of brain organization such as gene expression in cortical layers to receptor distributions, connections, morphology, and computation and function. These organization levels do not necessarily all show the same degree of granularity, and this is reflected in variations in the number and extent of areas depicted in each of these maps, not only when comparing brains from different species, but even within a given species. Elucidating the principles governing integration across hierarchical levels in order to understand how the different maps relate to one another, i.e. the “vertical translation”, is crucial to understand how structure subserves function, and constitutes one of the most exciting developments in neuroanatomy in the last decades (Mars et al. 2021).

Understanding how maps of different species relate to each other, i.e. the “horizontal translation”, is an equally challenging and fascinating endeavour and recently developed tools for relating maps between species will enable neuroscientists to address issues of homology and to equalize terminology across traditionally separated subfields of neuroscience (Mars et al. 2021). This horizontal translation is not only important from a comparative point of view. Since not all types of data can be obtained from any random species, a formal mapping between species is necessary to predict how unobtainable data could be present in any species of choice (Folloni et al. 2019; Tang et al. 2019). More importantly, this approach is crucial to facilitate translational neuroscience and bridge the gap between basic and clinical neuroscience, because grey matter structures with different phylogenetic origins are differentially susceptible to disease (Liu et al. 2021; van den Heuvel et al. 2019).

In this context, we decided to organize a special issue of *Brain Structure and Function* to bring together scientists aiming to increase our understanding of the relationship between the structural and functional segregation of the brain, although with different methods and in different species. This has resulted in a series of articles to be published in two volumes, i.e., “Towards multi-modal, multi-species brain atlases” parts one and two. The following sections provide a summary of the contents of part one.

The primate thalamus is the topic of a review by García-Cabezas et al. (2023), as well as of an original and a methodological article by He et al. (2022) and Pérez-Santos et al. (2023), respectively. The thalamus is a large subcortical diencephalic complex encompassing multiple structurally and functionally distinct nuclei which are organized into groups based on their developmental origin, structure, connectivity and the type of information they receive or transmit. García-Cabezas et al. (2023) provide a comprehensive overview of the historical developments leading to the two currently prevailing nomenclature and, more importantly, parcellation concepts. Importantly, they also critically discuss the advantages and disadvantages of each of these prevailing parcellation schemes, as well as the fact that they are each used by a different community of neuroscientists, namely those primarily involved in neurosurgical applications, and those using them for basic research purposes. In a complementary article, Pérez-Santos et al. (2023) propose a framework for future mapping studies of the primate thalamus which will ensure the production of methodologically reproducible data and apply a common terminology, thus facilitating comparability of results obtained at different institutions and future collaborative efforts. This framework is of enormous value given the expertise of the group led by Carmen Cavada, which over the last decades has published numerous detailed and highly cited studies on the distribution of subcortical neuromodulatory afferents to the thalamus (Cavada et al. 1995; García-Cabezas et al. 2007; Pérez-Santos et al. 2021; Rico and Cavada 1998). He et al. (2022) also pave the way for standardization of future studies through use of their Multi-modal-fused magnetic Susceptibility (MuSus-100) atlas nuclei in the human thalamus and basal ganglia. Their population based atlas is constructed from high resolution quantitative susceptibility mapping images, because they provide a higher contrast than that of T1-weighted or T2-weighted magnetic resonance images due to their higher sensitivity to iron and myelin (Bilgic et al. 2012; Langkammer et al. 2010) and to the fact that subcortical nuclei differ from each other and from their surroundings in their iron content.

Ikeda et al. (2022) and Rogers Flattery et al. (2023) apply largely diverging approaches to analyse the brains of the night monkey and the silver fox, respectively. Although these are not two of the most frequently used animal models, they provide the community with valuable insights into structure–function relationships in the brain. Rogers Flattery et al. (2023) present a beautiful dataset composed of annotated high resolution images of coronal thionin stained coronal sections through an entire *Vulpes vulpes* hemisphere accompanied by a mirror-reflected MRI volume re-sliced to match the plane of sectioning as closely as possible and which will serve as the neuroanatomical reference for the analysis of brain-behaviour relationships. This work also serves to demonstrate to the community what can be done with rare samples and how open data makes sure such resources can be of benefit to the wider scientific community. In contrast to this *post mortem* approach, Ikeda et al. (2022) applied high-resolution in vivo brain magnetic resonance imaging and comparative cortical surface T1w/T2w myeloarchitectonic mapping in the framework of the harmonized methodology provided by the non-human primate version of the Human Connectome Project pipeline to characterize the cortical organization of the night monkey and compare it to that of macaque and marmoset monkeys.

The availability of these new datasets and techniques enables researchers to look at patterns of anatomical organization that can form the basis of a phylogenetic understanding of variation across brains. One such overarching theory is Sanides’ (1970, 1962) Hypothesis on the Dual Origin of the Neocortex. Although not without its critics (Murray et al. 2017), this work has been very influential in guiding a number of cross-level studies of brain organization (Huntenburg et al. 2018) and increasingly is used to make comparisons across species. García-Cabezas et al. (2022) use differences in the shape of small and large cupcakes to visually demonstrate how their elaboration of Sanides’ (1962, 1970) Hypothesis on the Dual Origin of the Neocortex can explain the fact that the spatial proximity relationships between cortical types observed in the brains of rodentia are preserved in the brains of primates despite the neocortical expansion which has taken place in the latter order. Taking the comparative perspective to the extreme, this article is also a must-read for those interested in cupcakes.

Ultimately, much of the anatomical work on brain organization aims to help us understand its function. Passingham and Lau (2022) highlight that brain function can only be fully understood if results of functional imaging studies are analysed in the framework of the underlying structure. Each area is characterized by a distinct cellular and molecular composition, as well as a specific connectivity pattern, which conditions the kind(s) of input it receives, how this information is processed, and the areas to which this integrated/transformed information is forwarded.

We would like to conclude this editorial quoting Passingham and Lau (2022): “… the aim of neuroscience is not to simply label the brain, but to understand *how* it works.” We have tried with this special issue to provide novel insights into the relationship between the structural and functional segregation of the brain as well as highlight the new type of data and methods for between-level and between-species integration that are continuing to appear at a rapid pace. Finally, we believe that the original papers to appear in “Towards multi-modal, multi-species brain atlases: part two”, which is almost ready, are just as exciting as the ones published in this part of the Special Issue, and they will be of equal importance to further our understanding of the relevance of horizontal and vertical translation approaches in neuroscience.

## Data Availability

Data availability is not applicable to this article as no datasets were generated or analyzed.
